# The Combination of Three Components Derived from Sheng MaiSan Protects Myocardial Ischemic Diseases and Inhibits Oxidative Stress via Modulating MAPKs and JAK2-STAT3 Signaling Pathways Based on Bioinformatics Approach

**DOI:** 10.3389/fphar.2017.00021

**Published:** 2017-01-31

**Authors:** Fang Li, Yu Zhang, Donglin Zeng, Yu Xia, Xiaoxue Fan, Yisha Tan, Junping Kou, Boyang Yu

**Affiliations:** Jiangsu Key Laboratory of TCM Evaluation and Translational Research, Department of Complex Prescription of Traditional Chinese Medicine, China Pharmaceutical UniversityNanjing, China

**Keywords:** Sheng MaiSan, GRS, bioinformatics approach, myocardial ischemic diseases, oxidative stress, MAPKs signaling pathway, JAK-STAT signaling pathway

## Abstract

GRS is a drug combination of three components including ginsenoside Rb1, ruscogenin and schisandrin. It derived from the well-known TCM formula Sheng MaiSan, a widely used traditional Chinese medicine for the treatment of cardiovascular diseases in clinic. The present study illuminates its underlying mechanisms against myocardial ischemic diseases based on the combined methods of bioinformatic prediction and experimental verification. A protein database was established through constructing the drug-protein network. And the target-pathway interaction network clustered the potential signaling pathways and targets of GRS in treatment of myocardial ischemic diseases. Several target proteins, such as NFKB1, STAT3 and MAPK14, were identified as the candidate key proteins, and MAPKs and JAK-STAT signaling pathway were suggested as the most related pathways, which were in accordance with the gene ontology analysis. Then, the predictive results were further validated and we found that GRS treatment alleviated hypoxia/reoxygenation (H/R)-induced cardiomyocytes injury via suppression of MDA levels and ROS generation, and potential mechanisms might related to the suppression of activation of MAPKs and JAK2-STAT3 signaling pathways. Conclusively, our results offer the evidence that GRS attenuates myocardial ischemia injury via regulating oxidative stress and MAPKs and JAK2-STAT3 signaling pathways, which supplied some new insights for its prevention and treatment of myocardial ischemia diseases.

## Introduction

Myocardial ischemic disease is a public health concern with an increasing incidence that results in high morbidity and mortality worldwide (Yellon and Hausenloy, 2007). The therapeutic methods targeted at recovering blood flow to the ischemic myocardium are frequently applied in clinic. However, the process of reperfusion might trigger additional injury, as evidenced by an extension of the infarct size (Matsumura et al., 1998). It is well-known that oxidative stress is one of the main pathological factors of myocardial ischemic diseases (Lakshmi et al., 2009). Oxidative stress mediators, such as ROS release with high concentrations, could inhibit cell proliferation, augment myocardial apoptosis and increase infarct size, which significantly contribute to long-term mortality and chronic heart failure (Stanciu et al., 2000). Thus, exploring anti-oxidative stress agents is a therapeutic opportunity for ischemia diseases.

Disease state is considered as a systemic imbalance in the theory of traditional Chinese medicine (TCM), and TCM could adjust systemic imbalance through the network system of active components aiming at multiple pathways and targets (Li et al., 2007). TCM are drawing increased notice owing to their clinical curative efficacy for a long time. However, the obscure molecular mechanisms impeded their further development. Nowadays, the constant development of network pharmacology and improvement of biological network data have facilitated system level understanding of the interactions of genes, proteins, thus providing the novel approaches for illuminating the underlying mechanisms associated with the therapeutic efficacy of the active components of TCM (Li et al., 2011; Mei et al., 2012).

Network pharmacology could exert an influence at several respects in target and pathway identification and drug-target interactions illumination. Many literatures have successfully reported significant biological findings from these networks. Drug-target correlation networks are usually employed to interpret the mechanism of drug’s action (Gu et al., 2011). Meanwhile, the protein-disease and drug-drug network were also used to find out the relevance between the target proteins and diseases (Jiang et al., 2013). The theoretical algorithm named as CIPHER has been applied to discern the key targets and pathways (Wu et al., 2008). Network pharmacology has become the foundation stone for the elucidation of drugs’ mechanisms. Detecting the latent critical targets and signaling pathways for TCM against the diseases via bioinformatic approaches is time saving and efficient.

Sheng MaiSan is a famous traditional Chinese formula for the treatment of cardiovascular diseases in clinic (Zhang et al., 2008). Many main kinds of chemical ingredients, such as ginsenosides, homoisoflavonoids and lignans, have been reported to be responsible for the pharmacological activities of Sheng MaiSan (Wang et al., 2011). Developing a new type of modern drug combination with clear ingredients and controllable quality will be the new direction of TCM research. Meanwhile, it plays an essential role in speeding up the modernization and internationalization of TCM (Li et al., 2008). Recently, we screened a new drug combination (GRS, the proportion as 6:0.75:6) comprising three compounds, including ginsenoside Rb1 (Ginsenosides of Red ginseng-G), ruscogenin (Saponins of Radix ophiopogonis-R), and schisandrin (Lignans of Fructus schisandrae-S), which showed a significant cardioprotective effects in experimental studies (Kou et al., 2014). The proportion of the combination was optimized to achieve the highest efficacy, quality stabilization and clinical safety for research (Chu, 2014). However, the action mode and related signaling pathways against myocardial ischemic diseases of GRS are unknown. Network pharmacology could accelerate the comprehension and predict the potential critical targets and pathways, and the forecasting consequences might provide some clues for further experimental validation.

Therefore, to better understand how the combination GRS affects biological processes, the bioinformatic approach was implemented to illuminate the correlation of the targets and pathways of GRS. The active ingredients in combination GRS targeted numerous essential proteins with a high degree of specificity and with functional contributions to the main signaling pathways at the same time, which led to the new equilibrium of biological system against diseases. Then, the partial predictive results were verified in H9c2 cardiomyocytes stimulated by hypoxia/reoxygenation (H/R). H9c2 rat cardiomyocyte cell line have been widely used as a model for investigation of myocardial ischemic diseases (Zordoky and El-Kadi, 2007; Chou et al., 2010). And we induced injury by H/R, which is more physiologically relevant to mimic ischemia/reperfusion injury to cardiomyocytes *in vivo* (Shin et al., 2009). Our results will shed new light on the pharmacological mechanism of the combination derived from Sheng MaiSan and will facilitate an illustration of the detailed mechanisms of myocardial ischemic diseases in the network context, and lay the foundation for the further development of new drug with independent intellectual property.

## Materials and Methods

### Drugs and Reagents

GRS was a mixture of ginsenoside Rb1, ruscogenin and schisandrin (6:0.75:6). Schisandrin and ginsenoside Rb1 were obtained from Zelang Bio-Technology Co., Ltd (Nanjing, China). Ruscogenin was seperated in the laboratory and the purity was higher than 99%. Fetal bovine serum (FBS) was received from ScienCell (Carlsbad, CA, USA). Dulbecco’s modified Eagle medium (DMEM) was purchased from GIBCO/BRL (Life Technologies, Carlsbad, CA, USA). *N*-acetyl cysteine (NAC) was purchased from Sigma-Aldrich (St. Louis, MO, USA). 3-[4,5-Dimethylthiazol-2-yl]-2,5-diphenyltetrazolium bromide (MTT) was purchased from AMRESCO (Cleveland, OH, USA). The kits for determination of lactate dehydrogenase (LDH), the malondialdehyde (MDA) and the fluorescent kit for 2′, 7′-dichlorofluorescein diacetate (DCFH-DA) were obtained from Beyotime Institute of Biotechnology (Shanghai, China). Protease inhibitor, RIPA lysis buffer, and enhanced chemiluminescene (ECL) reagent were obtained from Vazyme Biotech (Nanjing, China). Antibody against GAPDH was purchased from Bioworld Technology (St. Louis Park, MN, USA), antibody against JNK, p-JNK, p38, p-p38, ERK1/2, p-ERK1/2 and Jak2, p-Jak2, Stat3, p-Stat3 were obtained from Cell Signaling Technology (Boston, MA, USA).

### Construction of the Protein Database

**FIGURE 1:**
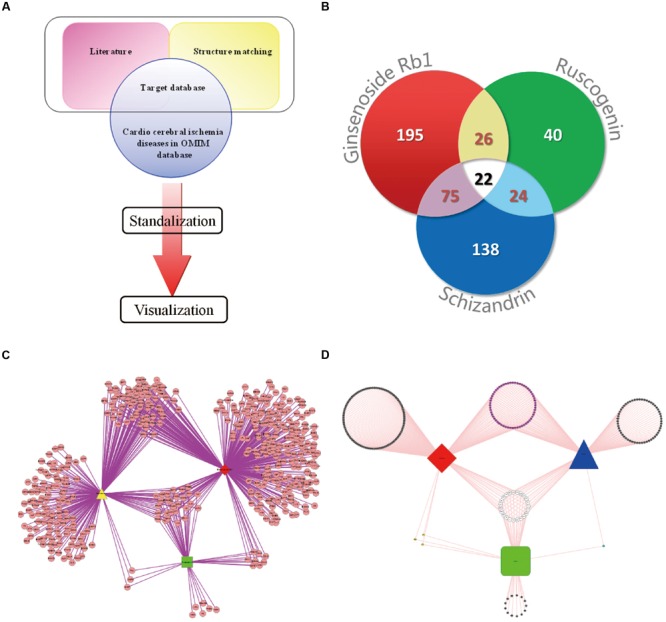
**Drug–target interaction network. (A)** The flowchart of model establishing. **(B)** Myocardial ischemic diseases related proteins information statistics. **(C)** The drug–target network of GRS. The red diamond represents ginsenoside Rb1; the yellow triangle represents schisandrin; the green square represents ruscogenin. **(D)** Compound-Myocardial ischemic diseases related protein network. The red diamond represents ginsenoside Rb1; the blue triangle represents schisandrin; the green square represents ruscogenin.

As reported in our previous studies (Tan et al., 2014; Li F. et al., 2015), the protein database was composed of two parts. **Figure 1A** depicts the process of this study. The first part of the database was composed of the known targets, which were retrieved from the literature database^1^. The search Mesh terms were assigned as “compound name” (#1 “ginsenoside Rb1,” #2 ruscogenin, #3 schizandrin, #4 #1 NOT #2 NOT #3, #5 #1 OR #2 OR #3). The proteins/genes which accorded with the Mesh terms were collected from the literatures^2^. The second part consisted the novel proteins related to the compounds with high affinity, which obtained from the PharmMapper database^3^ (Liu et al., 2010). Structures of the three components were established by Sybyl 6.9, and then were employed to all hydrogens and Gastiger-Huckel charges in Sybyl 6.9 in the Tripos force field in an implicit solvent condition (Li F. et al., 2015). The minimization steps included 1000 cycles of steepest descent until meeting convergent threshold of 0.05 kal-1Å-1 (Tan et al., 2014). Then the three compounds were imported into the PharmMapper human protein database, and the top 100 proteins were chosen. Meanwhile, the combined proteins/genes contained in the results were screened with the myocardial ischemic diseases from the OMIM, Genecards and DrugBank database. Protein–protein/gene–gene information included links to databases, such as BIND, BioGRID, DIP, HPRD, IntAct and MINT, which represented the major interactome for further bioinformatics analysis. The proteins/gene/GEO Profiles were standardized in the NCBI database entailed on *Homo sapiens*^4^.

### The Establishment and Analysis of the Network

The drug-target network was constructed using Cytoscape 2.8.3 (Cline et al., 2007). Nodes in the interactome corresponding to the genes and edges represented documented interactions in the visualization. Cytoscape could be download from the website http://cytoscape.org. Then the degree and betweenness of the ingredients were analyzed with the Centiscape module in Cytoscape. The most fundamental feature of the node was its degree, which showed how many links the node with other nodes. The betweenness and the degree were the node topological and centric indexes (Huang et al., 2009a; Li F. et al., 2015). Higher degree and larger betweenness nodes are most significant (Barabasi and Oltvai, 2004; Opsahl et al., 2010).

Network analysis was then subjected to GeneMANIA in Cytoscape, a plugin used for making fast and efficient gene function predictions. GeneMANIA algorithms have been exhibited to be as well as, or better than, in accuracy and speed compared with other gene function prediction algorithms. Furthermore, the GeneMANIA plugin was developed for the consideration and visualization of pathway interaction networks among the genes. The *Homo sapiens* database of GeneMANIA was previously downloaded from the internet, and then gene names were imported into the plugin, followed by selecting the right names and descriptions for them. Finally, the visualization network was represented as the pathway between the interaction genes.

On account of the high degree nodes from drug-target network, the proteins with little biological meanings in functional annotation chart through Biocarta analysis were eliminated (Huang et al., 2009b). Pathway analysis plays a vital part in detecting potential biological processes that the genes participate in. A variety of databases such as GeneGo, KEGG and Biocarta collect cured pathways (Apic et al., 2005). In our current study, the functional annotation clusters were divided into groups of similar annotations employing Biocarta functional annotation tool in accordance with the enrichment scores. Gene ontology (GO), another form of pathway, displays the cellular locations and physiological functions of multiple genes (Huang et al., 2009b). GO analysis were proceeded using the BINGO 2.4.4 plugin of Cytoscape 2.8.3. The genes were applied to cellular component; molecular function and biological process ontology file analysis.

### Cardiomyocytes Culture

Rat H9c2 cardiomyocytes was purchased from Shanghai Institute of Cell Biology, Chinese Academy of Sciences (Shanghai, China). The cells were grown in DMEM added with 10% FBS, 100 μg/mL streptomycin and 100 U/mL penicillin at 37°C in a humidified atmosphere containing 5% CO_2_.

### H/R Injury Model *In vitro*

The oxygen and glucose deprived (OGD) technique was established according to a previously described protocol (Rizvi et al., 2010). In current investigation, the OGD injury was conducted through supplementing none glucose DMEM and exposed to a hypoxic condition of 94% N_2_, 5% CO_2_ and 1% O_2_ at 37°C in a humidified N_2_/CO_2_ incubator and then in a standard incubator with normal atmosphere at 37°C. H9c2 cardiomyocytes were given with GRS (0.1–10 μg/mL) or *N*-acetyl cysteine (NAC, 500 μmol/L) during the hypoxia. At the beginning of reoxygenation, H9c2 cells were re-exposed to GRS and NAC.

### Cell Viability Assay and Measurement of LDH Activity

H9c2 cardiomyocytes growing exponentially were trypsinized and then approximately 8000 cells/well were seeded into 96-well plates. After the experimental treatment, cells were given with 0.5 mg/mL MTT for 3 h at 37°C. And then the medium was discarded and the formazan crystals was dissolved with DMSO. The absorbance was read at 570 nm with a reference wavelength of 650 nm. The LDH activity was then detected to further investigate the degree of cell injury. At the end of the experiment, the supernatants of culture medium were gathered. The LDH activity was measured at 490 nm following the manufacturer’s descriptions.

### Measurement of Malondialdehyde (MDA) and Intracellular ROS

The content of MDA was determined with assay kits according to the manufacturer’s instructions. In brief, cells were harvested by trypsinization and cellular extracts were lysed with RIPA lysis buffer. Then lysed cells were centrifuged and the supernatant were subjected to the measurement of MDA levels and the protein contents. MDA contents were normalized to milligram protein. Additionally, ROS levels were detected using 2′, 7′-dichlorofluorescein diacetate (DCFH-DA). The cardiomyocytes were first incubated with DCFH-DA (10 μmol/L) for 30 min, then washed with PBS and visualized by fluorescence microscopy.

### Western Blot Analysis

As reported (Guan et al., 2013), the H9c2 cardiomyocytes were lysed with RIPA lysis buffer added with protease inhibitor. The cell lysates were then centrifuged and protein concentrations were calculated using a BCA protein assay kit (Beyotime Institute of Biotechnology, Shanghai, China). The equal amounts of protein (40 μg) were separated by denaturing 12.5% SDS-PAGE and transferred onto PVDF membranes. The membranes were then blocked with 3% BSA and incubated with primary antibodies against JNK, p-JNK, p38, p-p38, ERK1/2, p-ERK1/2, Jak2, p-Jak2, Stat3, p-Stat3 and GAPDH (dilution 1:1000, 1:800, 1:1000, 1:800, 1:1000, 1:1000, 1:1000, 1:500, 1:1000, 1:500, 1:8000, respectively) overnight at 4°C. Then membranes were probed with peroxidase conjugated secondary antibody with a 1: 8000 dilution. The antigen-antibody complexes were then tested with ECL reagent and visualized by ChemiDoc^TM^ MP System (Bio-Rad), and analyzed using Image Lab^TM^ Software (version 4.1, Bio-Rad).

### Statistical Analysis

All experiments were performed in triplicate and the data are expressed as the mean ± SEM. Student’s two-tailed *t*-test for comparison between two groups and one-way analysis of variance (ANOVA) were employed for statistical analysis. Dunnett’s test was applied when the data involved more groups. *P* < 0.05 was defined as significant.

## Results

### Drug-Target Interaction Network

The characteristics of the drug-target network were shown in **Figure 1B** and **Table 1**. Then, we constructed the network of potential targets of the components in GRS by cytoscape, as shown in **Figure 1C**. Further, we excluded little biological meaning proteins with Biocarta functional annotation analysis. According to the correlation between proteins with biological meaning, we re-built the protein targets network related to myocardial ischemic diseases of the combination GRS, as illustrated in **Figure 1D**. Obviously, the drug-target network was dense and the combination GRS targeted a great deal of proteins. Moreover, the drug-target network results displayed that a single component could act on multiple proteins, while different composition could play a role in the same protein. The link between each component and proteins, protein and protein are close, and the overall correlations are evenly distributed.

**Table 1:** The general network properties of the drug-target interaction network.

Number of nodes	Number of edges	Average number of neighbors	Network centralization	Characteristic path length	Network heterogeneity
268	373	2.784	0.725	2.582	5.250

### Evaluation of the Important Proteins

**FIGURE 2:**
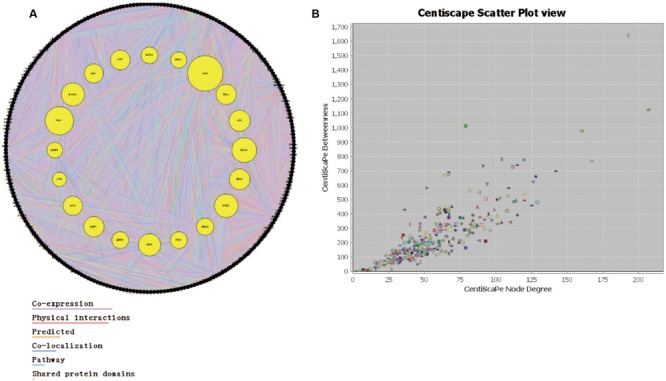
**The analysis of interaction network. (A)** The protein–protein interaction network. **(B)** The relationship between node betweenness and degree distribution.

**FIGURE 3:**
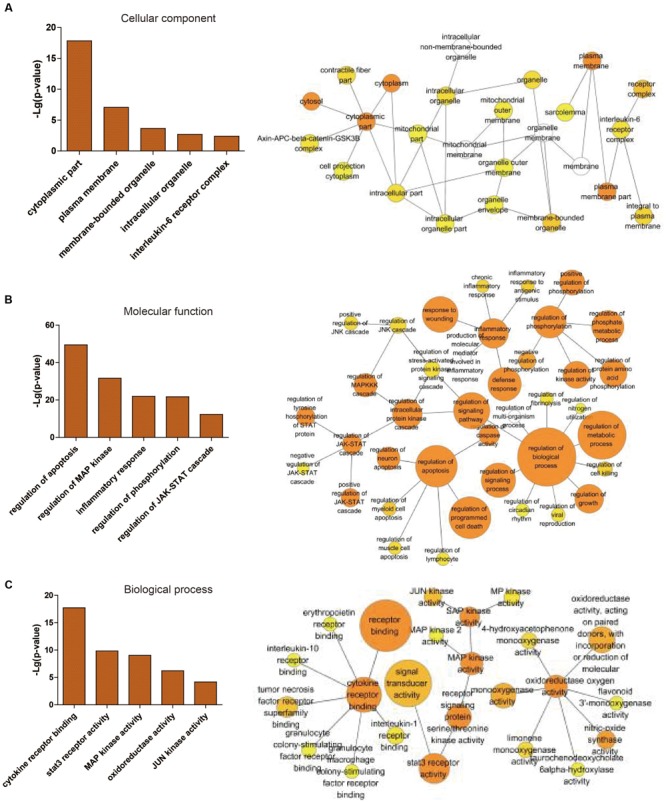
**Statistical analysis of gene ontology metadata.** Statistical analysis of the gene ontology classification was performed using the BiNGO plugin in cytoscape. The left picture is a graphical representation of top 5 scored -lg (*P*-value) significantly enriched ontologies, presented as the cellular component **(A)**, molecular function **(B)** and biological process **(C)**. The right picture is the enlarged figures including different numbers of top ontologies in the analysis. The orange-marked ontologies in the left are contained in the corresponding right figures.

Through the information technology analysis and forecast of the relationship between protein and protein, we constructed the protein–protein interaction network of the combination targets related with myocardial ischemic disease (**Figure 2A**). Then, we selected the essential proteins which have the higher connection degree and larger betweenness from the network (**Figure 2B**) for becoming the candidate key proteins, such as NFKB1, JUN, EGF, STAT3, CDH5, HIF1A, FOS, TNF, APP, ESR1, MAPK14, SLC2A1, TP53, MAPK8, all of which were associated with MAPKs, JAK-STAT, oxidative stress and NF-κB signaling pathways and so on (**Table 2**). Furthermore, we found the protein, which was significantly enriched by analysis of genetic background of the protein in the network, such as STAT3 acceptor, MAP kinase and JNK kinase. The graphical representations of the relative positions of the terms in the GO hierarchy were displayed in **Figures 3A–C**, with the extent of importance showed according to color. The major cellular component ontologies (**Figure 3A**) were cytoplasmic part, plasma membrane, membrane-bounded organelle, intracellular organelle and interleukin-6 receptor complex. The terms such as regulation of apoptosis, regulation of MAP kinase, inflammatory response, regulation of phosphorylation and regulation of JAK-STAT cascade were significantly enriched in the molecular function ontologies (**Figure 3B**). In addition, the enriched biological process ontologies were managed by cytokine receptor binding, stat3 receptor activity, MAP kinase activity, as well as oxidoreductase activity and JUN kinase activity (**Figure 3C**).

**Table 2:** Proteins information of high degree connection and correlation.

Gene symbol	Betweenness	Degree
NFKB1	1643.686	193
JUN	1125.209	207
EGF	1010.55	79
STAT3	976.7627	160
CDH5	778.7818	104
HIF1A	775.6348	120
FOS	767.6101	167
TNF	738.1665	115
APP	731.193	91
ESR1	728.0267	112
MAPK14	697.1574	142
SLC2A1	672.8005	67
TP53	660.8239	128
MAPK8	622.0975	126
SRC	604.3359	94
HSP90AA1	581.0109	109
PRL	580.4675	52
CSF3	562.9149	82
CD14	548.6541	88
MAPK1	537.9401	120
MAPK3	523.9574	109
ITGB2	506.0382	103
MMP9	505.1719	93
ATF3	500.4878	101
AKT1	494.9877	114
IL6	494.3673	120
CAV1	486.5094	113
EGR1	484.6549	114
IL8	482.2064	129
NFKBIA	458.8095	125

### The Pathway Enrichment Analysis

**FIGURE 4:**
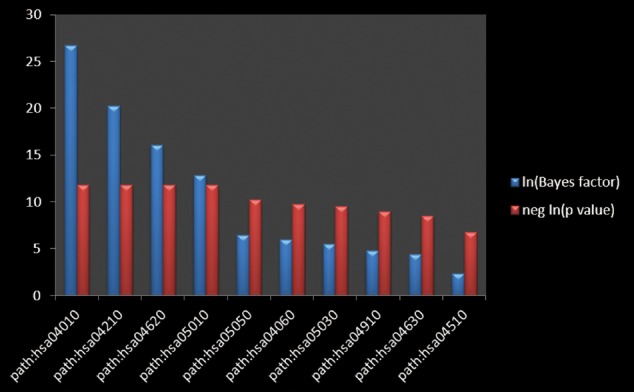
**The pathway enrichment analysis.** The Bayes factor quantifying the amount of evidence supporting the hypothesis that the annotation is associated with your gene list. The negative logarithm of the *p*-value calculated from the Bayes factor, calculated using a Fisher’s exact test.

The top 10 main functional annotation clusters were displayed in **Table 3** and **Figure 4** ranked by the Biocarta functional annotation cluster tool. The functional annotation cluster divided the categories of similar annotations following their enrichment scores. The relevant protein terms in the highest scored cluster were related with the MAPK signaling pathway. The second main pathways related to apoptosis. And the rest clusters on comprised pathways related to Toll-like receptor, Alzheimer’s diseases, Dentatorubro pallidoluysian atrophy, Cytokine–cytokine receptor interaction, Amyotrophic lateral sclerosis, Insulin, JAK-STAT and Focal adhesion signaling pathways. On the basis of the enrichment scores, the MAPK signaling pathway was the most possible candidate for GRS function aiming at myocardial ischemic diseases.

**Table 3:** The representive signaling pathways for annotation cluster of GRS.

Annotation cluster	Signaling pathway
hsa04010	MAPK signaling pathway
hsa04210	Apoptosis
hsa04620	Toll-like receptor signaling pathway
hsa05010	Alzheimer’s disease related pathway
hsa05050	Dentatorubro pallidoluysian atrophy
hsa04060	Cytokine–cytokine receptor interaction
hsa05030	Amyotrophic lateral sclerosis
hsa04910	Insulin signaling pathway
hsa04630	JAK-STAT signaling pathway
hsa04510	Focal adhesion

### Experimental Validation

**FIGURE 5:**
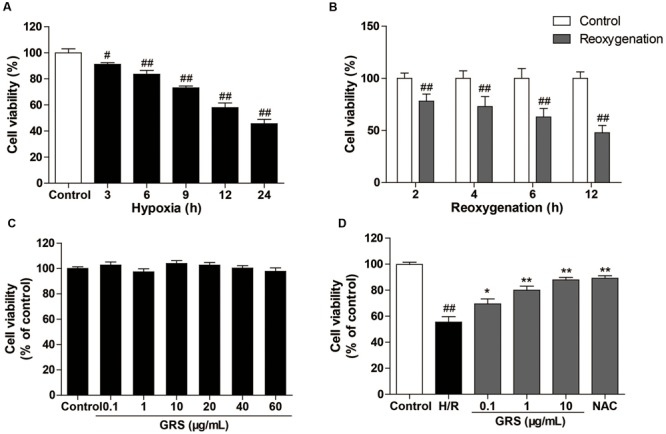
**Effects of GRS on H/R induced H9c2 cardiomyocytes injury.** H9c2 cardiomyocytes were exposed to hypoxia followed by reoxygenation. Then the cell viability was detected by the MTT assay described in method. **(A)** Effect of hypoxia on H9c2 cell viability. **(B)** Effect of reoxygenation on H9c2 cell viability. **(C)** Effect of GRS on H9c2 cell viability. **(D)** H9c2 cells were treated with GRS followed by H/R and cell viability was determined by MTT assay. Results were obtained from three independent experiments and were presented as mean ± SEM. ^#^*P* < 0.05, ^##^*P* < 0.01 vs. control group without H/R, ^∗^*P* < 0.05, ^∗∗^*P* < 0.01 vs. group treated with H/R alone.

**FIGURE 6:**
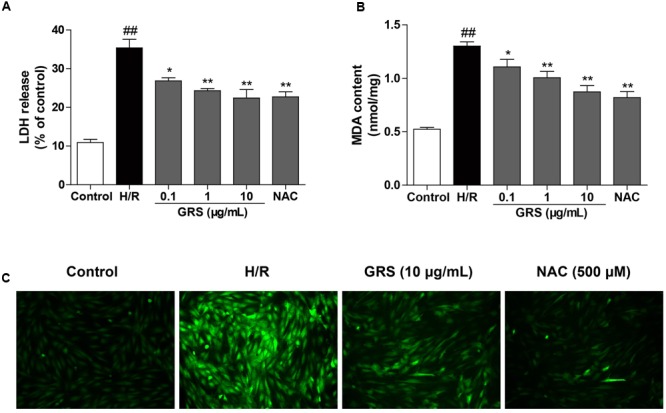
**Effects of GRS on LDH release (A)**, MDA content **(B)** and intracellular ROS **(C)** in H9c2 cardiomyocytes subjected to H/R injury. H9c2 cardiomyocytes were treated with GRS at the concentration of 0.1–10 μg/mL and then exposed to hypoxia of 6 h followed by 6 h reoxygenation. LDH release and MDA content were measured by assay kit. ROS levels were determined using 2′, 7′-dichlorofluorescein diacetate (DCFH-DA, Molecular Probes). NAC was used as a positive control drug. Results were obtained from three independent experiments and were presented as mean ± SEM. ^##^*P* < 0.01 vs. control group without H/R, ^∗^*P* < 0.05, ^∗∗^*P* < 0.01 vs. group treated with H/R alone.

We first examined the effects of different condition of H/R injury on H9c2 cardiomyocytes viability. As shown in **Figure 5A**, hypoxia caused a time-dependent decrease in cell viability. Then, the cells were exposed to hypoxia for 6 h, followed by different times of reoxygenation. Adequate cell injury extent was observed at 6 h of reoxygenation (**Figure 5B**). Moreover, we detected the viability of cardiomyocytes after the treatment with GRS and GRS at concentrations of 0.1–60 μg/mL did not markedly influence cardiomyocytes viability (**Figure 5C**). Then three concentrations (0.1, 1 and 10 μg/mL) were chosen for further detection. H9c2 cardiomyocytes exposed to H/R (H 6 h/R 6 h) led to a decrease in cell viability, while given with 0.1–10 μg/mL GRS and 500 μM positive control drug NAC maintained cell viability (**Figure 5D**). As LDH release was an accepted sign for cell injury, the release of LDH in culture medium was determined. LDH release markedly increased after H/R injury as compared to control group, whereas treatment with 0.1–10 μg/mL GRS and 500 μM NAC markedly inhibited the release of LDH (**Figure 6A**). These results indicated that GRS had an obvious protective effect against H/R-mediated damage in H9c2 cardiomyocytes.

As a significant product of lipid peroxidation, MDA indirectly reveals the generation of intracellular ROS. When cells were subjected to H/R injury, the content of MDA increased remarkably. Whereas, the concentration of MDA significantly decreased after treatment with 0.1–10 μg/mL GRS and 500 μM NAC (**Figure 6B**). Meanwhile, compared to the control group, ROS production was markedly increased in the H/R group. While cells treated with GRS showed a substantial reduction in ROS levels (**Figure 6C**).

**FIGURE 7:**
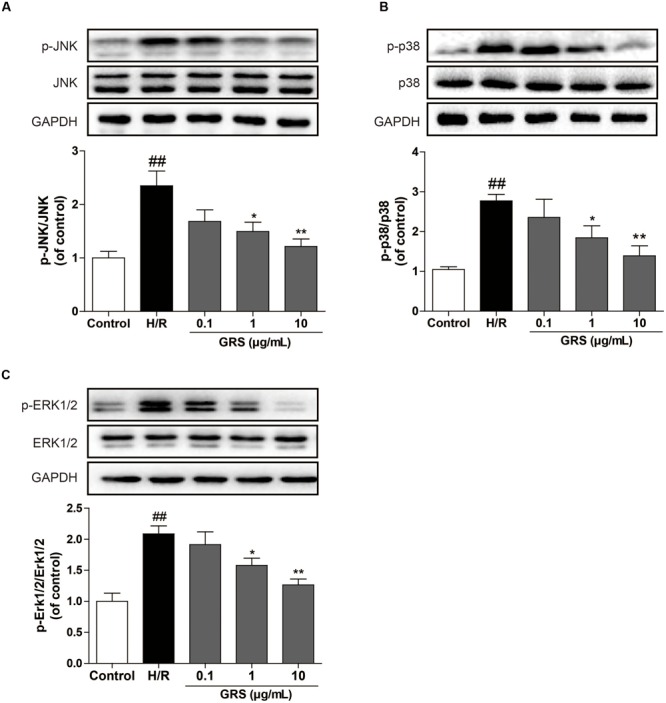
**Effects of GRS on MAPKs signaling pathway in H/R induced H9c2 cardiomyocytes.** H9c2 cardiomyocytes were treated with GRS at the concentration of 0.1–10 μg/mL and then exposed to hypoxia of 6 h followed by 2 h reoxygenation. Protein expressions were detected by western blot. **(A)** Protein expressions of JNK and p-JNK using GAPDH as the loading control. **(B)** Protein expressions of p38 and p-p38 using GAPDH as the loading control. **(C)** Protein expressions of ERK1/2 and p-ERK1/2 using GAPDH as the loading control. Results were obtained from three independent experiments and were presented as mean ± SEM. ^##^*P* < 0.01 vs. control group without H/R, ^∗^*P* < 0.05, ^∗∗^*P* < 0.01 vs. group treated with H/R alone.

As shown in **Figure 7**, we evaluated whether GRS regulated the MAPKs signaling pathway to validate the results from the target-pathway interaction network analysis. The Western blotting results revealed that H/R injury significantly increased phosphorylation of JNK, p38 and ERK1/2 in H9c2 cardiomyocytes, indicating that H/R injury activated MAPKs pathway. While 1–10 μg/mL GRS could down-regulate phosphorylation of JNK, p38 and ERK1/2, and there was no difference in total JNK, p38 and ERK1/2 protein abundance among all groups detected.

**FIGURE 8:**
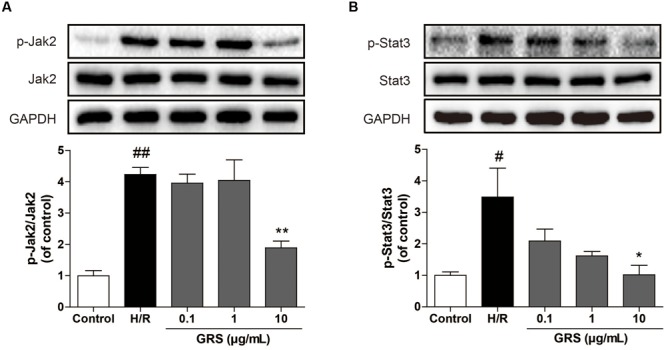
**Effects of GRS on Jak-Stat signaling pathway in H/R induced H9c2 cardiomyocytes.** H9c2 cardiomyocytes were treated with GRS at the concentration of 0.1–10 μg/mL and then exposed to hypoxia of 6 h followed by 2 h reoxygenation. Protein expressions were detected by western blot. **(A)** Protein expressions of Jak2 and p-Jak2 using GAPDH as the loading control. **(B)** Protein expressions of Stat3 and p-Stat3 using GAPDH as the loading control. Results were obtained from three independent experiments and were presented as mean ± SEM. ^#^*P* < 0.05, ^##^*P* < 0.01 vs. control group without H/R, ^∗^*P* < 0.05, ^∗∗^*P* < 0.01 vs. group treated with H/R alone.

In addition, we further investigated whether GRS could regulate the JAK-STAT signaling pathway. As shown in **Figure 8**, we found that GRS at the concentration of 10 μg/mL could inhibit the phosphorylation of JAK2 and STAT3 under H/R conditions, suggesting that GRS might play a role in protecting myocardial ischemia injury through the inactivation of JAK2-STAT3 signaling pathway.

## Discussion

Reperfusion-induced oxidative stress is a key contributor to myocardial ischemia-reperfusion (MI/R) injury by inducing a cascade of pathological changes, such as DNA damage, mitochondrial dysfunction and protein aggregation, which in turn lead to cardiomyocytes apoptosis (Sinha et al., 2013). MI/R-induced redox aberrance results from excessive ROS generation and antioxidant system impairment (Chinda et al., 2013). Disrupt balance between oxidants and antioxidants lead to uncontrolled myocardial injury. Our study provided evidence that the combination GRS inhibited H/R-induced H9c2 cardiomyocytes injury via potent antioxidant effect as inferred by reduced MDA levels and ROS generation. Whereas, the especial pathway and potential mechanisms has yet to be explored.

As we all know, the complexity of components and obscure mechanisms hinder the development and internationality of TCM in the world. In recent years, network pharmacology has provided an efficient approach for the underlying molecular mechanisms elucidation of TCM. We applied the network of drug-target and target-pathway interaction to discover the potential targets and pathways of the combination of three bioactive components derived from Sheng MaiSan to decipher its mechanism in the treatment of myocardial ischemic diseases.

We found that 30 of nodes with high-degree were existed in the highest annotation cluster pathway. Many pharmacological investigations exhibited that the transcription factor NF-κB exerts an essential part in cell proliferation and serves as an important modulator of the inflammatory process. The activity suppression of NF-κB could protect myocardial ischemia reperfusion induced heart injury (Liang et al., 2014). STAT3 has been indicated to be essential for cardioprotective effects such as preservation of LV functional reserve and perfused capillary density, decrease of apoptotic cell death and reduction of infarct size (Smith et al., 2004). Moreover, MAPK participated in the regulation of cellular responses to a variety of stimuli, such as mitogens and pro-inflammatory cytokines. Mitogen-activated protein kinases 3, 8 and 14 regulate proliferation, gene expression, cell survival, apoptosis and many other cell functions (Pearson et al., 2001; Li G. et al., 2015). The function mechanism of these important proteins might be that they influence the integral network instead of one target alone. The overall network would be disrupt in a pathological state once the critical targets are pulled out from the network. On the contrary, the pathological condition would become normal when very critical proteins aimed at.

Gene ontology has been extensively employed to interpret gene products in any organism as the ontology for designating structural controlled vocabulary terms (Li F. et al., 2015). And GO is composed of three separate ontologies, including cellular component, molecular function and biological process, which describe the attributes of gene products or gene product group. The three annotation levels were in accordance with signal pathways existed in the functional annotation clusters, which were correlated with the regulation of MAP kinase and the JAK-SATA cascade. This elucidated the action mechanism from the perspective of GO.

Biocarta functional annotation tool was applied to group the functional annotation clusters with similar annotations on the basis of the enrichment scores. The proteins in each groups would be in high relevance with each other. The most essential protein terms in the topmost scored cluster are correlated with the MAPKs signal pathway. Intracellular MAPK (mitogen-activated protein kinase) signaling cascades exert an important part in the pathogenesis of cardiovascular diseases (Suchal et al., 2016). MAPKs are divided into three subgroups in general: p42/p44 extracellular signal-related kinases (ERK1/2), p38 MAPK, and c-Jun N-terminal protein kinase (JNK) (Muslin, 2008). Reactive oxygen species generated due to ischemia-reperfusion injury could activate MAPKs signaling pathways (Gao et al., 2015). When MAPKs were activated, a variety of different substrate proteins were phosphorylated, including essential modulatory enzymes, apoptosis factors and cytoskeletal proteins, through influencing the phosphorylation state of the substrate proteins (Zhou et al., 2002).

Myocardial ischemia injury activated p38 MAPK, with the transcription factors and protein phosphorylation, increasing adhesion molecule expression and granulocyte activation, eventually leading to myocardial necrosis and apoptosis of large area (Ma et al., 1999). Meanwhile, the oxide produced by reperfusion injury could result in JNK MAPK activation, and then activate a variety of transcription factors and trigger programmed cell death (Ferrandi et al., 2004). Various studies have demonstrated that targeted inhibition of p38/JNK protect the myocardium from ischemia and reperfusion injury by reducing oxidative stress, inflammation, cardiomyocyte apoptosis, metabolic abnormalities and by maintaining cytoskeletal architecture (Li et al., 2014; Gao et al., 2015). In general, the ERK cascade is regarded to participate both cell proliferation and survival. Studies had previously demonstrated that activation of ERK1/2 at the time of myocardial reperfusion confer powerful cardioprotection, indicating a potential amenable pharmacological target for cardioprotection (Armstrong, 2004). While latest investigations have verified that, the persistent activation of ERK is also participated in the process of apoptosis (Plotkin et al., 2005; Sun et al., 2012). GRS might produced an interlinked network, thus, these building blocks of the body could exert effect as a whole. In the current research, we detected the efficacy of GRS on the expression and activation of MAPKs signaling pathway in H/R-induced H9c2 cardiomyocytes, confirming the forecasting consequences and offering an illustration of the molecular mechanisms of myocardial ischemia diseases. As an active component of GRS, ginsenoside Rb1 has been reported to alleviate the ischemia-reperfusion injury through the inhibition of p38 MAPK and JNK MAPK (Li et al., 2012, Li G. et al., 2015). We found that ruscogenin suppressed mouse neutrophil activation through the inhibited phosphorylation of p38 MAPK, ERK1/2 and JNK (Lin et al., 2015). Schisandrin could also exert anti-inflammatory effects via inhibition of JNK and p38 MAPK activities in a RAW 264.7 macrophage cell line (Guo et al., 2008).

Due to the large number of literatures with the correlation between the three components in combination GRS and MAPKs signaling pathway, thus, we further investigate whether the combination GRS derived from Sheng MaiSan exerts cardioprotective effects through other novel signaling pathway. Then we focused on the Janus kinase (JAK)-signal transducers and activators of transcription (STAT) signaling pathway based on the former predicted results. JAK-STAT pathway is a stress-responsive mechanism that delivers signals from the cell surface to the nucleus, hence, regulating gene expression, which plays an essential role in the mechanism of heart diseases with mediating myocardial cell growth, survival and apoptosis (Bolli et al., 2003). Latest researches have illustrated that ischemia and reperfusion injury in myocardium lead to quick activation of this pathway and activation intervention of this pathway induces the recovery in cardiac function (Mascareno et al., 2001). JAK2 and STAT3 are central component of cardioprotection (Bolli et al., 2011). In our study, we observed that GRS could markedly inhibit the activation of JAK2-STAT3 signaling pathway. The JAK-STAT signaling pathway is significant for cytokine receptor signaling associated with inflammatory responses. Our investigations demonstrate that the activation of the JAK2-STAT3 signal pathway is possible involved in the emergence and progression of systemic inflammatory response. Therefore, the activation inhibition of JAK2-STAT3 signaling pathways might due to the reduction of inflammatory response. Moreover, the drugs might exerted different effects in different detecting time points and further investigations are needed to explore the underlying protection mechanism in depth. To the best of our knowledge, this is the first study, which illustrates that the regulation of the JAK2-STAT3 pathway exerts a vital part in the pharmacological action of GRS.

## Conclusion

The combination of three components derived from Sheng MaiSan appears effective in mitigating H/R-induced H9c2 cardiomyocytes injury via suppression of oxidative stress. Then we applied bioinformatics approach to predict the potential molecular mechanisms of the combination GRS against myocardial ischemia diseases based on an integrated target-pathway interaction network. According to this network, we constructed the molecular targets and signaling pathways correlations between bioactive components and myocardial ischemia diseases. Then we confirmed partial results through experiments in cell model. These outcomes illustrated that the bioinformatics methods were effective in predicting and illuminating the interaction between drug actions and complex diseases. Due to these combined methods, the MAPKs and JAK2-STAT3 signaling pathways were discerned and validated to exert an significant part in the combination GRS mechanism against myocardial ischemia diseases. Further investigations need to proceed more researches of the efficacy of active components on other signaling pathways and their interactions. In addition, the H9c2 rat cardiomyocytes used in our research could not precisely represent cardiac myocytes though it was extensively used in the investigation of cardiovascular diseases. And application of primary cultured cardiac myocyte is in progress to sustain the cardioprotective effect of GRS.

## Author Contributions

All the authors provided important intellectual content, reviewed the content and approved the final version for the manuscript. FL, YZ, and XF performed the experiments; FL and YT analyzed the data; FL, DZ, and YX prepared figures; FL and XF wrote the manuscript; JK and BY reviewed and edited the manuscript.

## Conflict of Interest Statement

The authors declare that the research was conducted in the absence of any commercial or financial relationships that could be construed as a potential conflict of interest.

## Footnotes

1 http://www.ncbi.nlm.nih.gov/pubmed/

2 http://www.ncbi.nlm.nih.gov/mesh/

3 http://59.78.96.61/pharmmapper/

4 http://www.ncbi.nlm.nih.gov/geoprofies/
